# Techniques for Thin-Walled Element Milling with Respect to Minimising Post-Machining Deformations

**DOI:** 10.3390/ma13214723

**Published:** 2020-10-22

**Authors:** Magdalena Zawada-Michałowska, Józef Kuczmaszewski, Stanisław Legutko, Paweł Pieśko

**Affiliations:** 1 Faculty of Mechanical Engineering, Lublin University of Technology, 20-618 Lublin, Poland; j.kuczmaszewski@pollub.pl (J.K.); p.piesko@pollub.pl (P.P.); 2 Faculty of Mechanical Engineering, Poznan University of Technology, 60-965 Poznań, Poland; stanislaw.legutko@put.poznan.pl

**Keywords:** deformations, milling, thin-walled elements, aluminium alloys, machining

## Abstract

The paper examines the impact of selected machining techniques and the semi-finished product technological history on deformations of thin-walled elements made of EN AW-2024 T351 aluminium alloy after milling. The following techniques have been implemented: High Performance Cutting, High Speed Cutting, conventional finishing (CF) and combinations of these techniques. As for the semi-finished product technological history, the rolling direction has been analysed. It has been assumed that it can be relevant in relation to the cutting tool feed direction and, in consequence, exert considerable impact on the stress, as well as deformation following machining. The interest in this issue proceeds from significant challenges faced by the industry, particularly in the aerospace sector. The analysis of results obtained has shown that milling in the direction perpendicular to the rolling direction results in larger deformations than milling in the parallel direction. Additionally, it has been revealed that applying a correctly selected machining technique makes it possible to minimise post-machining deformations of thin-walled elements.

## 1. Introduction

The growing interest in issues related to thin-walled element machining results from their broad-range applications in various industry sectors, in particular, in the aerospace and automotive industries. In aircraft, thin walls are included in the design of fuselage, control surface, on-board equipment and body, etc. [1,2,3].

Increasingly frequently, the term “integral (structural) thin-walled elements” is used. Their characteristics comprise a uniform design, relatively high rigidity and low weight in comparison to their overall dimensions and a high strength to empty weight ratio [1,4,5].

Currently, integral elements are manufactured mostly using state-of-the-art 5-axis machining centres by means of high-performance machining techniques, such as: HSC (High Speed Cutting) [6] and HPC (High Performance Cutting). They are very often produced using monolithic plates made of light metal alloys (aluminium, titanium) [7]. In high-performance milling of such elements, chips amount to as much as 95% of the semi-finished product weight [4,5,8,9,10,11,12,13].

One of the main problems encountered during thin-walled element machining are elastic and plastic deformations. Elastic deformations result in geometry errors, vibrations and machined surface quality deteriorations. They are caused by the phenomenon of the workpiece sagging under the cutting edge impact. Plastic deformations result in shape errors and generate residual stresses which are difficult to eliminate from the element’s surface layer and cause permanent alterations of its size and shape. In industrial practice, among others, interoperational heat treatment, e.g., relief annealing is used to remove residual stresses. Due to economic reasons, such operations are being eliminated, as they generate additional costs and increase the production time significantly [4,9,14,15,16,17,18].

In the industry, problems related to large-sized thin-walled elements and their significant deformations occurring directly after removing the clamping forces are quite common. It is assumed that the reasons for the occurring deformations can be as follows [19,20,21]:

- post-machining residual stresses introduced during machining;
- residual stresses introduced during semi-finished product manufacturing (i.e., technological history effect);
- residual stresses introduced during heat treatment.

Thus, the end condition of residual stresses in the surface layer, after machining, is important [19,21].

During machining, plastic deformation of the workpiece material occurs around the cutting tool tip, which, after removing the factor that has caused it, creates compressive residual stresses in the surface layer. Additionally, machining processes generate heat (among others, as a result of friction) that results in considerable temperature differences in the cutting zone, and thermal stresses exceeding the material yield point, which, in consequence, causes tensile residual stresses in the surface layer. It should also be taken into account the fact that structural changes and resulting volume changes occur in a material [22,23,24].

It is assumed that, after machining, mainly mechanical stresses (pressure) and thermal stresses (temperature), operating in the opposite direction, dominate in the surface layer. In reality, both factors impact the residual stress condition; however, their intensity may vary. The mechanical model mostly refers to machining, and the thermal model is characteristic for abrasive machining and HSC. It must be highlighted that residual stresses occur at the depth of a few decimals of a millimetre and depend on numerous factors, e.g., machining parameters, tool geometry and machining methods [25,26,27,28,29,30,31].

Thin-walled aircraft components are often designed as pocket-like elements mostly made of wrought aluminium alloys. In such cases, rolled plates are usually used as semi-finished products, having different structural and mechanical properties in particular directions, in relation to the rolling direction (anisotropy phenomenon). It is related to the accumulation of residual stresses resulting from the semi-finished product technological history. During machining, the balance of the underlying stress condition is disrupted and new stresses are introduced, which results in the occurrence of undesirable deformations [19,21,32,33,34,35,36].

Thin-walled element deformations are difficult to forecast, as, apart from residual stresses, they result from a number of other factors, including: temperature, clamping force, cutting force, tool geometry, etc. [13,19,30,37,38,39,40,41]. 

Additionally, common problems related to thin-walled element machining include [15,42,43,44]:

- maintaining good surface quality;
- difficulties in ensuring required dimensional and shape accuracy;
- the presence of self-excited vibrations (“chatter”) disturbing the machining process stability.

More and more often, cutting fluids are being eliminated from the machining process, mainly due to their negative impact on the environment. Methods such as Minimum Quantity Cooling Lubrication (MQCL) and Minimum Quantity Lubrication (MQL) become alternatives to traditional cooling methods that are now widely used, e.g., during manufacturing of the thin-walled elements [45,46].

The enhancement of dimensional and shape accuracy of manufactured thin-walled elements is obtained by the application of the following machining error minimisation methods [10,16,17,47,48]:

- correct milling strategy selection;
- increase in cutting speed *v_c_* (HSC);
- rationalisation of cutting parameters (in particular: feed per tooth *f_z_* and milling width *a_e_*) aimed at decreasing the component of cutting force perpendicular to a wall being machined.

It must be stressed that the correlation between the selection of correct procedures aimed at minimising post-machining errors and types of machined materials is also significant.

The choice of an optimum thin-walled element machining strategy mostly depends on the ratio between the machined wall height and its thickness. Due to the above, three cases can be identified [17,48,49]:

- low height to thickness ratio < 15:1—where separate milling of each wall side in non-overlapping passes is recommended;
- moderate height to thickness ratio < 30:1:
  ○ milling on a constant level—alternate machining at a constant depth of cut *a_p_* of both side walls, also in non-overlapping passes;
  ○ milling at a difference of levels—alternate milling of both side walls with non-overlapping levels between consecutive passes; the depth of cut *a_p_* at the first pass should be *a_p_*/2;
- high height to thickness ratio > 30:1—Where it is recommended to change the sides and apply the “christmas tree” routine in order to achieve the wall thickness setpoint in stages.

Generally, as a rule, thin wall machining processes are planned in such a manner that non-machined material locally supports the material being machined [50]. The number of tool passes mostly depends on the milled wall dimensions and assumed depth of cut *a_p_*. It is also recommended that the duration of cutting edge contact with a workpiece should be decreased in thin-walled element machining. It can be achieved by applying increased cutting speed *v_c_*, a low *a_p_*/*a_e_* coefficient and feed in range of 0.02–0.05 mm/tooth. Additionally, it is advisable to provide an 0.1–1 mm allowance for finishing [16,17,46].

Authors [36,51] specify the following thin-walled element deformation minimisation methods:

- cutting parameter optimisation;
- tool geometry optimisation;
- tool path optimisation;
- designing a special clamping device;
- simultaneous workpiece wall machining on either side.

In machining, particularly in relation to aircraft components, the “8:1 rule” also applies, i.e., it is recommended that 1 mm thick walls should be milled using the depth of cut *a_p_* equal to 8 mm or less. Other applicable guidelines specify that it is advisable to leave the stiffening ribs (mostly on the side opposite to the machined surface) which must be removed at the end of the process. The guidelines presented are based on available industrial experience gained using the trial and error method [51,52].

The simulations performed in the paper [37] have shown that, in order to improve the dimensional and shape accuracy and machined thin-walled element surface quality, variable cutting parameters for each layer must be applied.

Recommendations regarding thin-walled element manufacturing on their entire height have also become quite common. In such cases, tools with high rigidity and correct geometry must be used [44].

During thin-walled plate machining, the tool penetration process should be started from the middle, using the helical interpolation. Next, the machining should be continued outwards from the assumed starting point. While milling a surface with the opposite side already machined, minimum pressure should be applied, and tools with a minimum number of cutting edges should be used [46].

Summing up the literature analysis conducted, one may conclude that the majority of papers include theoretical scientific considerations and numerical simulations forecasting the size of occurring deformations. They fail to present application-related information and technological guidelines that might facilitate solving significant problems encountered by the machine industry, related to the presence of thin-walled element deformations following machining processes. Only a few papers attempt to perform experiment-based verification of the theoretical models created, and identify the possibilities of practical applications in techniques employed to manufacturing such elements.

The aims of the paper are to indicate the milling technique that ensures the smallest post-machining deformations of thin-walled elements made of EN AW-2024 T351 aluminium alloy and to determine the influence of the technological history of the semi-finished product on the value and type of deformations as well as to define the recommended minimum thickness of the surface layer after the rolling that has to be removed during pre-machining.

## 2. Materials and Methods

**Figure 1:**
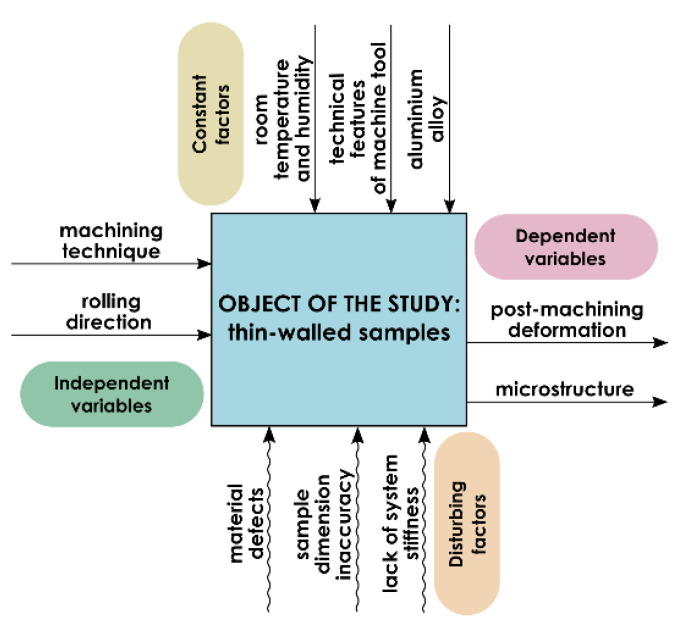
Research object model.

The research object model, including independent and dependent variables as well as constant and disturbing factors, is presented in Figure 1.

**Figure 2:**
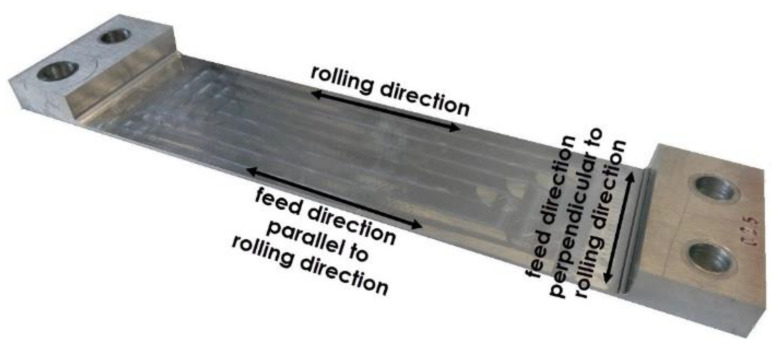
View of a sample after milling, with the relation between the cutting tool feed direction and milling direction indicated.

The research object comprised thin-walled samples made of EN AW-2024 T351 aluminium alloy of the following dimensions: 45 mm × 210 mm × 10 mm (Figure 2). The machining technique and rolling direction were assumed as the independent variables. The analysed dependent variables included post-machining deformation and the surface layer microstructure. The constant factors were machine tool technical features, laboratory temperature and humidity as well as the material type. The disturbances included material defects, sample dimension inaccuracy and lack of system (machine tool–clamping device–workpiece–cutting tool) rigidity.

The research was performed in relation to five machining techniques, i.e.,

- High Performance Cutting;
- High Performance Cutting and conventional finishing (CF);
- High Performance Cutting and High Speed Machining;
- High Speed Cutting;
- High Speed Cutting and conventional finishing (CF).

The relation between the cutting tool feed direction and the rolling direction, defined as the technological history effect, was another independent variable subject to examination. Two variability levels were assumed:

- cutting tool feed direction perpendicular to rolling direction (perpendicular direction);
- cutting tool feed direction parallel to rolling direction (parallel direction).

Figure 2 shows a view of a thin-walled sample after milling, with the relation between the cutting tool feed direction and milling direction indicated. The created pocket dimensions were 45 mm × 160 mm, and the bottom thickness was 1 mm. The sample, in addition to the part subject to analysis, also has left ends for the samples clamping during machining. The holes were used to jig samples in the clamping device (ensuring uniform clamping conditions). The adoption of such geometric solution ensures repeatability of the clamping in the entire series of samples and eliminates the influence of the clamping forces on the deformation process. In the study, the research was carried out on flat samples, it was the most favourable solution to achieve the goals.

The paper deliberately assumed that the surface on the side opposite to the machined surface would remain unmachined, which is not typical in the processes of manufacturing elements from this type of semi-finished products. The goals of the solution are:

- the assessment of the impact of the rolling technological history on the deformation of thin-walled elements after machining;
- defining the minimum allowance of the rolled surface layer to be removed in elements manufactured from such semi-finished products.

The research was based on samples cut out of a rolled plate made of the EN AW-2024 wrought aluminium alloy, in the T351 condition. It is one of the most popular multi-component aluminium alloys which, due to its high strength, is widely used in the aerospace industry to manufacture, e.g., wing sheathing and airframe fuselage. The EN AW-2024 alloy is characterised with good machining properties and is supplied following various thermal treatment variants which exert considerable impact on its mechanical properties. However, its disadvantages include low corrosion resistance and limited weldability. The chemical composition of EN AW-2024 aluminium alloy is presented in Table 1.

The EN AW-2024 T351 aluminium alloy has the following properties [54]:

- density, *ρ* = 2.78 g/cm^3^;
- Young’s modulus, *E* = 73 GPa;
- tensile strength, *R_m_* = 469 MPa;
- offset yield strength, *R_p0.2_* = 324 MPa;
- Brinell hardness, 120 HB.

Machining was performed with the Avia VMC 800 HS vertical machining center (FABRYKA OBRABIAREK PRECYZYJNYCH AVIA S.A., Warsaw, Poland) with the Heidenhain iTNC 530 control (Heidenhain, Traunreut, Germany). 

**Figure 3:**
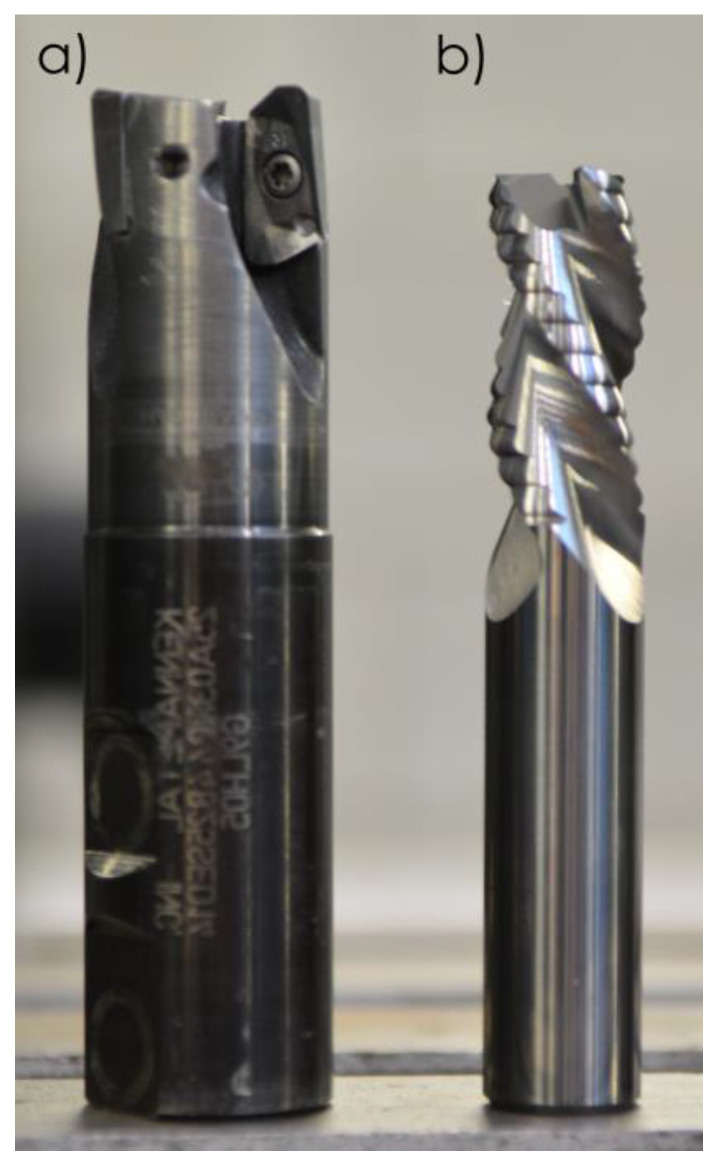
Tools used in studies: (**a**) Kennametal indexable milling cutter; (**b**) Sandvik monolithic milling cutter.

Three milling cutters were used in the studies, i.e.,

- indexable milling cutter by Kennametal (25A03R044B25SED14) (Kennametal, Pittsburgh, PA, USA) with correctly selected cutting inserts (EDCT140416PDFRLDJ)—used for HPC (Figure 3a);
- monolithic carbide milling cutter by Sandvik (R216.33-16040-AC32U) (Sandvik, Stockholm, Sweden) without a protective coat—used for HSC and conventional finishing (CF) (Figure 3b).

Due to the fact that the Sandvik milling cutter is dedicated to roughing, the explanation is needed. The geometry of the Sandvik tool used in the form of the Kordell geometry, apart from the chip breaker, does not differ from the milling cutter for finishing. In this case, HSC and conventional machining with small depths of cut were used, so the cutter working part with chip breaker was not used.

Tool selection was based on the current range of milling cutters employed in the aluminium alloy machining (ISO N group), particularly taking into account possibility of their use for High Performance Cutting and High Speed Cutting. Specifications of tested milling cutters are shown in Table 2.

According to the tool materials, KC410M and H10F are a trade mark used by the manufacturers. Kennametal reports that it is a carbide coated with TiB2 protective coating applied by the PVD method and Sandvik gives information that it is a tungsten carbide without any protective coating. 

The cutting parameter values used for each machining technique analysed are presented in Table 3.

The cutting parameters were selected both on the basis of tool manufacturers’ recommendations and authors’ own experience. Additionally, the flood cooling with an aqueous solution of cooling oil based on MobileCut 230 coolant concentrate (Mobil industrial, Spring, TX, USA) was used. 

**Figure 4:**
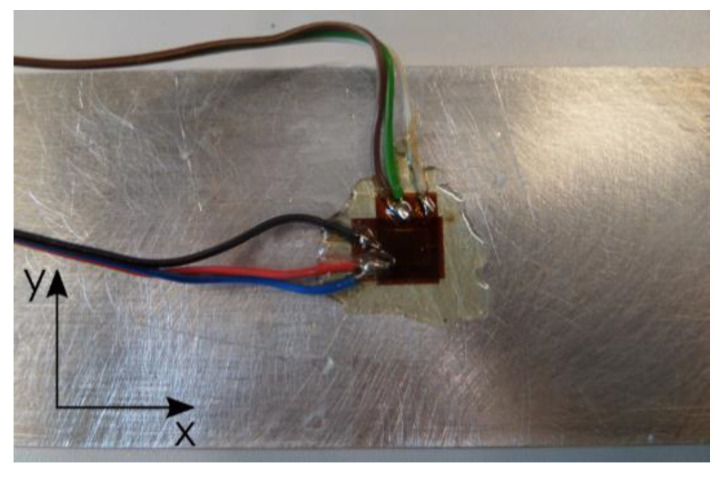
Foil strain gauges glued on sample surfaces.

The deformations were studied by means of the strain gauge method using Tenmex TF-5-2x foil strain gauges (Tenmex, Łodź, Poland). Two individual strain gauges were glued to examined sample surface in its central part, according to following directions (Figure 4):

- x—parallel to the longer sample edge (longitudinal strain gauge);
- y—perpendicular to the longer sample edge (transversal strain gauge).

The selection of the place was related to the prediction of the greatest strains in this part, while one strain gauge in each direction was enough to measure these strains.

The process of attaching strain gauges to the examined sample surfaces was conducted in line with a standard procedure. The LOCTITE 401 cyanoacrylate adhesive (Henkel, Düsseldorf, Germany) was used plus the M-COAT A AIR DRYING POLYURETHANE COATING (Micro-Measurements, Wendell, NC, USA) to protect strain gauges against impact exerted by a coolant and other external factors. The measurement system used in the examination included strain gauges which, by means of soldered conductors, were connected with SCMSG120 adapters, HBM 1-MX840A universal measurement amplifier (HBM, Darmstadt, Germany), optoelectronic connector and computer with the CatmanEasy V35.1 DAQ PROJECT software installed to process, analyse and visualise the measurement results obtained. The examination was repeated five times for each configuration analysed.

**Figure 5:**
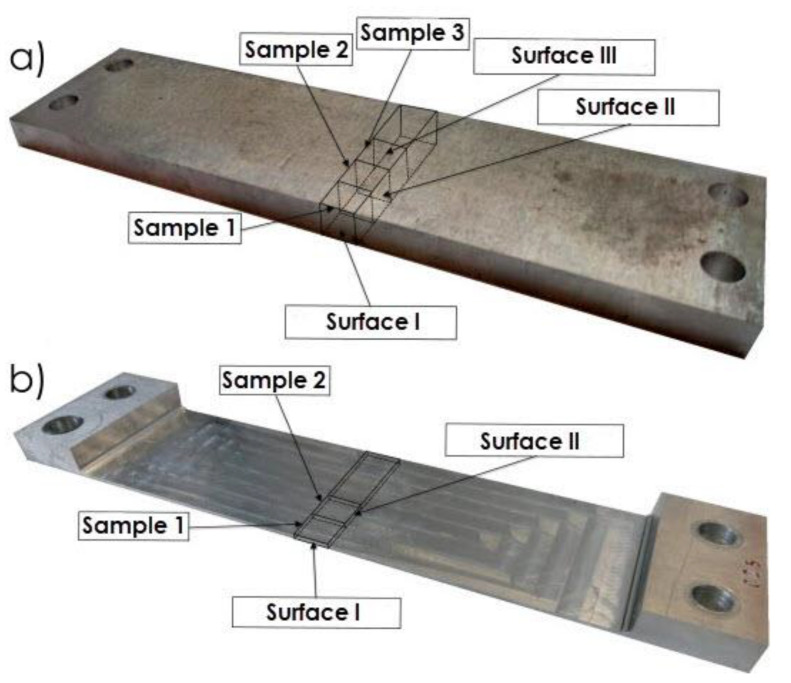
Locations where samples used to prepare metallographic specimens were taken, including highlighting of the examined surfaces: (**a**) sample prior to machining; (**b**) sample after machining.

The study consisted of examining the surface layer microstructures, both before and after milling, taking into account all configurations analysed. The Nikon Epiphot inverted metallographic microscope (Nikon, Tokio, Japan) with the ToupView software installed was used to examine the structures. The locations where samples used to prepare metallographic specimens were taken, including highlighting of the examined surfaces, are presented in Figure 5a (sample prior to machining) and Figure 5b (sample after machining).

The aim of the microstructures was to compare the depth of textured surface layer after the rolling and the milling for various techniques analysed in the study as well as to define the recommended minimum thickness of the surface layer after the rolling that has to be removed during pre-machining.

Metallographic specimens were prepared following the standard procedure applied for aluminium alloys. Firstly, the samples were ground with sandpaper of increasing grit number, polished with the MetaDi diamond suspension (Buehler, Lake Bluff, IL, USA) and OP-S NonDry aluminium oxide suspension (Struers, Cleveland, OH, USA). After polishing, surfaces were etched using Mi2Al solution.

## 3. Results

**Figure 6:**
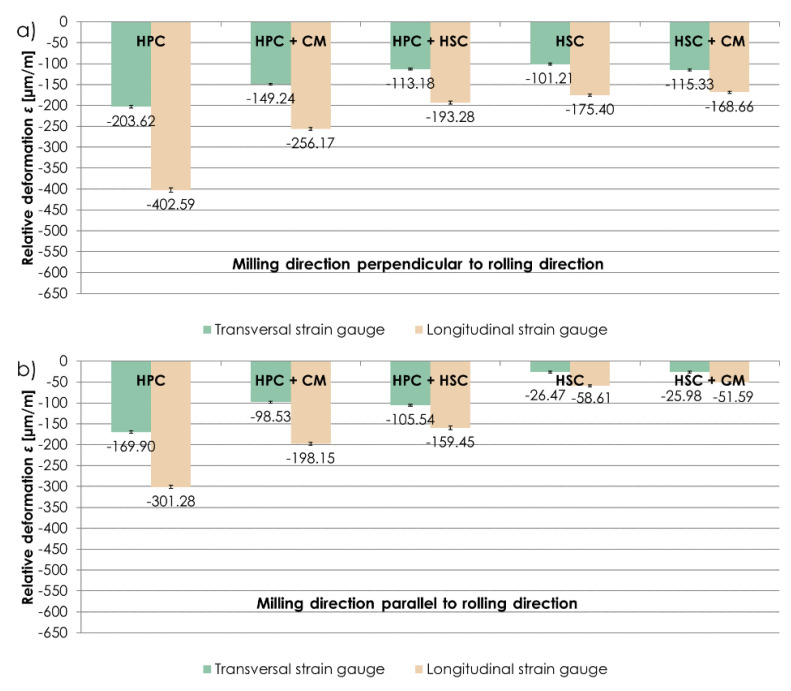
Relative deformations *ε* obtained using the analysed machining techniques, and with (**a**) perpendicular, (**b**) parallel relationship between the milling direction to the rolling direction.

The examination concentrated on relative deformation *ε* sign and values during the stabilisation phase, after removing a sample from a clamping device and stabilising it to the ambient temperature level. Figure 6 presents relative deformations *ε* obtained for the machining techniques examined, in which the milling direction was respectively perpendicular and parallel to the rolling direction. Relative deformations *ε* were expressed in µm/m, showing by how many µm units an element was deformed along a 1 m long section. The obtained results showed that, regardless of the relation between the milling direction and rolling direction, larger relative deformations *ε* were detected on longitudinal strain gauges. “-” means that strain gauges were stretched. Due to the noticeable relationship between the relative deformation *ε* values obtained on strain gauges glued perpendicularly and in parallel to the longer sample edge, it was decided to consider the results of relative strain *ε* from the longitudinal strain gauge, in the further part of this paper. For milling perpendicular to the rolling direction, maximum relative deformations *ε* were obtained using the HPC technique, i.e., *ε* = −402.59 µm/m, and minimum deformations were obtained using the HSC technique combined with conventional finishing (CF), i.e., *ε* = −168.66 µm/m (almost 60% less in relation to HPC). In the case of milling parallel to the rolling direction, an analogous relationship was observed. The largest relative deformations *ε* were present following HPC, i.e., *ε* = −301.28 µm/m, and the smallest ones following HSC combined with conventional finishing (CF), i.e., *ε* = −51.59 µm/m (over 80% less in comparison with HPC).

**Figure 7:**
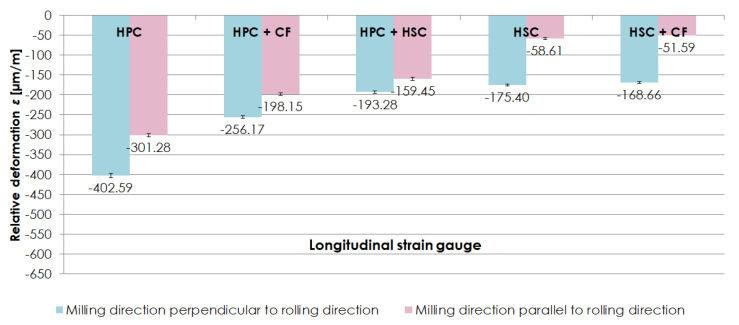
Comparison of relative deformations *ε* obtained on longitudinal strain gauges, depending on the relationship between the milling direction and rolling direction.

Figure 7 shows a comparison of relative deformations *ε* obtained on longitudinal strain gauges, depending on the relationship between the milling direction and rolling direction. While analysing the results, it was established that samples milled perpendicularly to the rolling direction were subject to larger relative deformations *ε* than samples milled in parallel to the rolling direction. With HPC, combination of HPC with conventional finishing (CF) and combination of HPC with HSC, the difference between relative deformations *ε* was approximately 30% (in relation to the parallel direction). In the case of HSC and combination of HSC with conventional finishing (CF), relative deformations *ε* were 200% larger with milling in the direction perpendicular to the rolling direction than in the parallel direction.

The analysis of the results obtained in the relative deformation *ε* examination performed on thin-walled samples made of EN AW-2024 aluminium alloy in the T351 condition enabled to conclude that, regardless of the milling technique analysed, higher relative deformations *ε* were obtained after milling in the direction perpendicular to the rolling direction. A possibility to reduce relative deformations *ε* by applying a correct machining technique was also observed.

**Figure 8:**
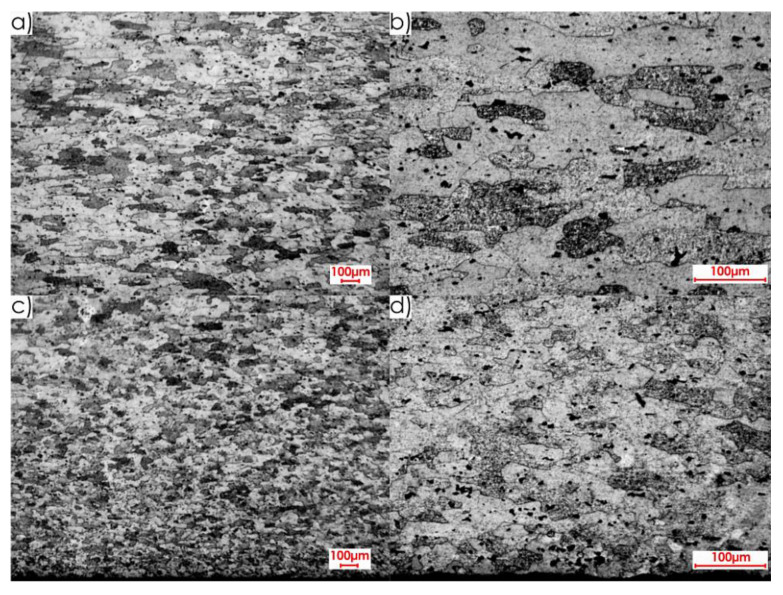
Microstructure of surface I for perpendicular rolling direction: core: (**a**) 2.5× magnification, (**b**) 20× magnification; surface layer after rolling: (**c**) 2.5× magnification, (**d**) 20× magnification.

**Figure 9:**
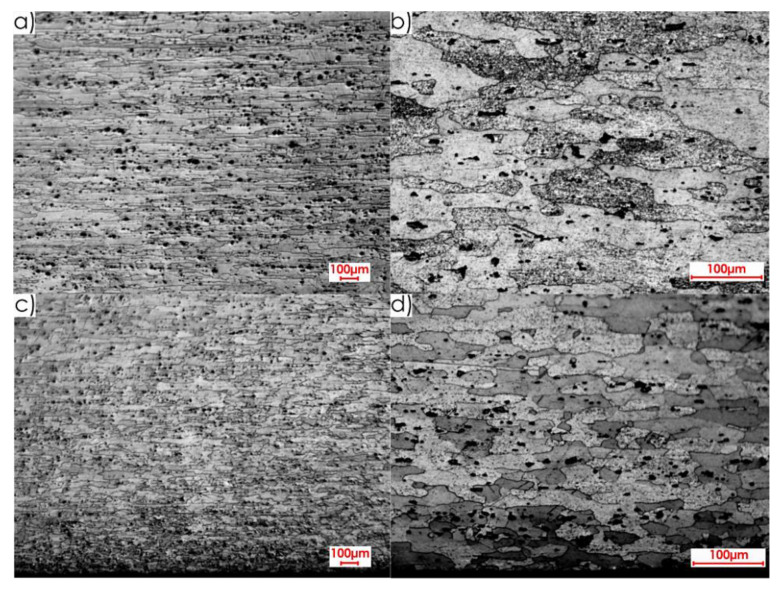
Microstructure of surface I for parallel rolling direction: core: (**a**) 2.5× magnification, (**b**) 20× magnification; surface layer after rolling: (**c**) 2.5× magnification, (**d**) 20× magnification.

During the first stage of the microstructure observations, samples prior to the milling process were taken into account. The first step was to analyse the surfaces designated as I, II and III in Figure 5a. Figure 8 presents views of the microstructure of surface I for samples cut out in the direction perpendicular to the rolling direction, at the following magnification: 2.5× and 20×, and Figure 9 shows analogous views for the parallel direction. Thus, both the core structure and surface layer resulting from rolling were taken into account.

**Figure 10:**
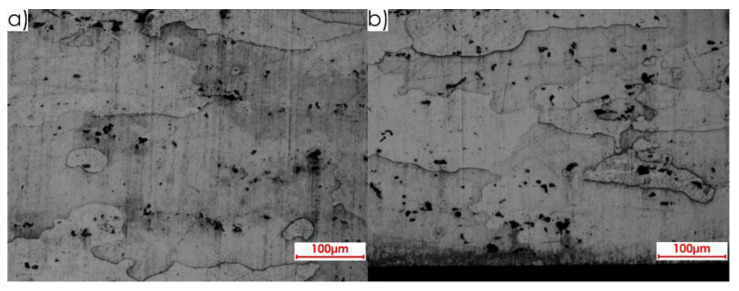
Microstructure of surface II for perpendicular rolling direction (20× magnification): (**a**) core, (**b**) surface layer after rolling.

**Figure 11:**
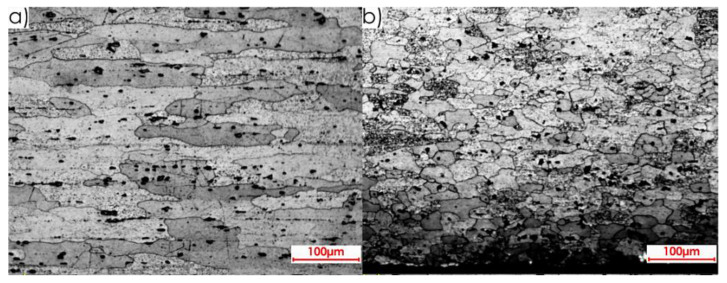
Microstructure of surface II for parallel rolling direction (20× magnification): (**a**) core, (**b**) surface layer after rolling.

The microstructures of both core and surface layer after rolling observed on surface II for the perpendicular and parallel rolling direction, at 20× magnification, are shown in Figure 10 and Figure 11.

**Figure 12:**
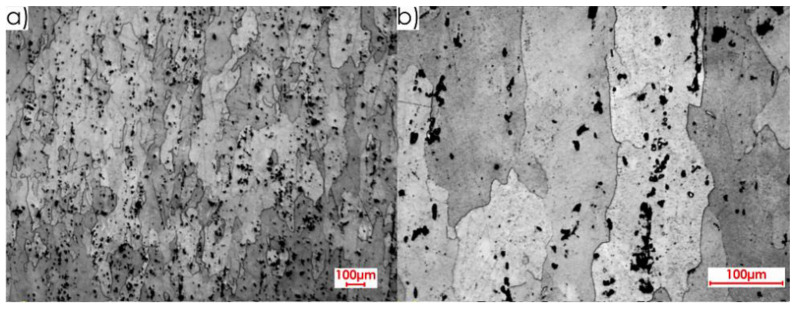
Microstructure of surface III for perpendicular rolling direction: (**a**) 2.5× magnification, (**b**) 20× magnification.

**Figure 13:**
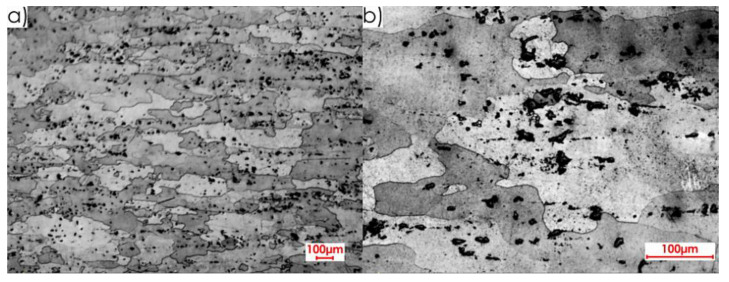
Microstructure of surface III for parallel rolling direction: (**a**) 2.5× magnification, (**b**) 20× magnification.

Figure 12 and Figure 13 show the microstructures of surface III for the perpendicular and parallel rolling direction obtained at the following magnification: 2.5× and 20×.

**Figure 14:**
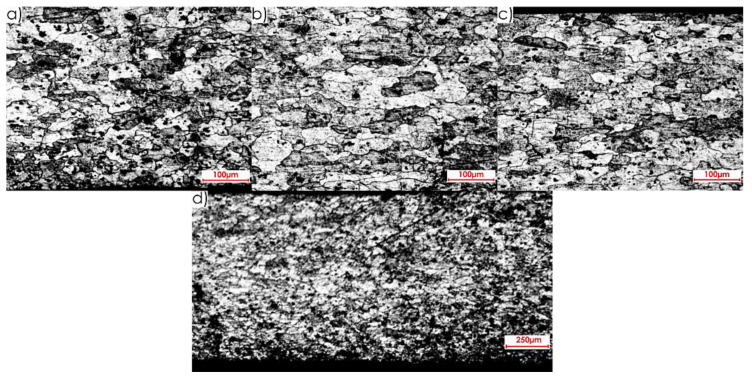
Microstructure after machining using the HPC technique for surface I and perpendicular rolling direction: 20× magnification: (**a**) surface layer after rolling, (**b**) core, (**c**) surface layer after HPC; 2.5× magnification: (**d**) all sample structure view.

**Figure 15:**
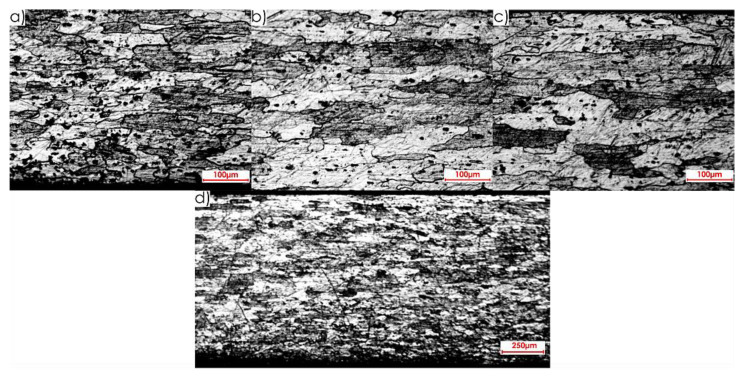
Microstructure after machining using the HPC technique for surface I and parallel rolling direction: 20× magnification: (**a**) surface layer after rolling, (**b**) core, (**c**) surface layer after HPC; 2.5× magnification: (**d**) all sample structure view.

Next, images of the microstructure of thin-walled samples (1 mm thick) were analysed after the milling, taking account of the techniques studied and the rolling directions considered. Focus was directed at surfaces designated as I and II in Figure 5b. Due to slight differences in the microstructure for individual machining techniques, results are presented only for one selected technique, i.e., HPC. Figure 14 presents the microstructure of surface I after milling with the HPC technique and the perpendicular rolling direction obtained at the following magnification: 2.5× and 20×, whereas Figure 15 shows views for the parallel direction. The structures of both the core and surface layer resulting from rolling and HPC were taken into account.

**Figure 16:**
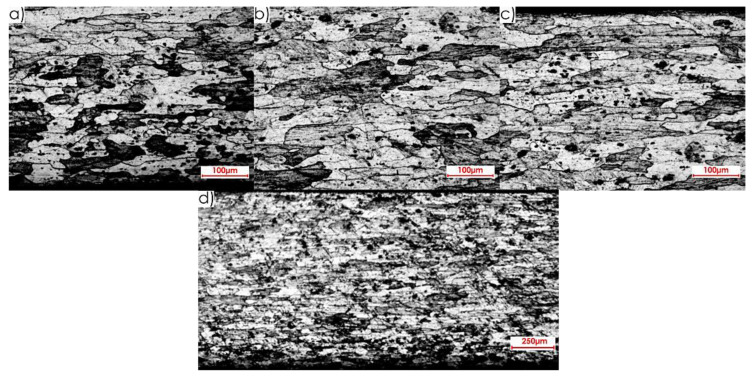
Microstructure after machining using the HPC technique for surface II and perpendicular rolling direction: 20× magnification: (**a**) surface layer after rolling, (**b**) core, (**c**) surface layer after HPC; 2.5× magnification: (**d**) all sample structure view.

**Figure 17:**
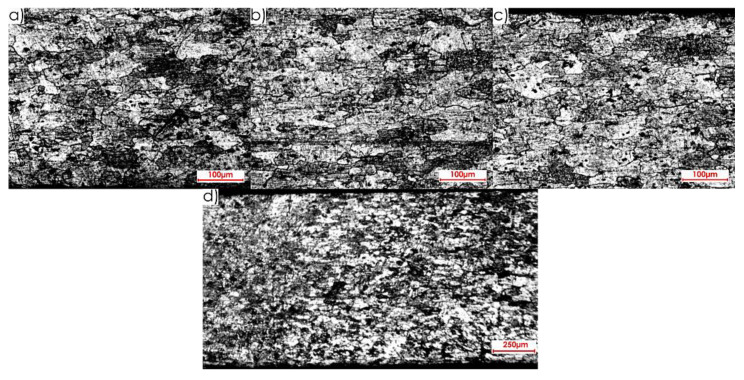
Microstructure after machining using the HPC technique for surface II and parallel rolling direction: 20× magnification: (**a**) surface layer after rolling, (**b**) core, (**c**) surface layer after HPC; 2.5× magnification: (**d**) all sample structure view.

Views of the microstructure of both the surface layer and the core, after High Performance Cutting for surface II and the studied rolling directions (perpendicular and parallel), obtained at the applied magnifications (20× and 2.5×, respectively), are shown in Figure 16 and Figure 17.

On the basis of the microstructure examinations performed, a clear difference between the surface layer and the core, after both milling and rolling, was identified. Additionally, diverse structures after machining and plastic forming were observed. In the case of rolling, longer and flattened grains were obtained, and the whole textured zone had a thickness of approximately 0.4 mm, while after milling, shorter and slightly smaller grains were received, and the textured zone was approximately 0.2 mm. It was defined on the basis of the observation of the microstructures in an approximate way. Hence the thickness of the textured zone is 50% higher after rolling than after milling. 

In addition, it is recommended to conduct the experiment in a version with the use of a pre-machining consisting in the removal of the textured surface layer formed after rolling (0.4 mm), as it is highly likely that it will minimise post-machining deformations. 

## 4. Conclusions

The completed research and analysis of obtained results have enabled to formulate the following conclusions:

- The removal of forces clamping a thin-walled workpiece in a clamping device creates a new dimension and shape balance under the influence of the residual stresses resulting from both rolling and milling, which results in significant deformations, invisible during the machining.
- It is possible to minimise post-machining deformations of thin-walled elements made of EN AW-2024 T351 aluminium alloy by employing a correct milling technique.
- Larger relative deformations *ε* were observed on longitudinal strain gauges.
- The relation between the milling direction and rolling direction exerts a significant impact on occurring deformations. Regardless of the milling technique, larger relative deformations *ε* were obtained after milling in the direction perpendicular to the rolling direction.
- Different microstructures were observed after milling and rolling. In the case of rolling, elongated grains were obtained, and the whole textured zone had a thickness of approximately 0.4 mm, while after machining, shorter and slightly smaller grains were obtained, and the textured zone was approximately 0.2 mm (50% less than after rolling).

## Figures and Tables

**Table 1:** The chemical composition of EN AW-2024 aluminium alloy: own prepared based on [53].

Chemical Composition, %										
Si	Fe	Mg	Cu	Mn	Zn	Cr	Zr+Ti	Ti	Other	Al
≤0.5	≤0.5	1.2–1.8	3.8–4.9	0.3–0.9	≤0.25	≤0.1	≤0.2	≤0.15	≤0.15	Rest

**Table 2:** Specifications of milling cutters used [55,56].

Symbol	Kennametal 25A03R044B25SED14	Sandvik R216.33-16040-AC32U
Material	KC410M ^1^	H10F
Number of teeth, *z*	3	3
Working part diameter *d*, mm	25	16
Overall length *L*, mm	101	92
Maximum depth of cut *a_pmax_*, mm	14.6	32
Clamping part diameter *d*, mm	25	16

^1^ Cutting insert material.

**Table 3:** Cutting parameter values for specific machining techniques.

Cutting Parameters	Techniques							
	HPC	HPC + CF		HPC + HSC		HSC	HSC + CF	
		HPC	CF	HPC	HSC		HSC	CF
Depth of cut *a_p_*, mm	4.5	4.3	0.4	4.3	0.4	0.956; 0.4 *	0.956	0.4
Milling width *a_e_*, mm	18.75	18.75	12	18.75	12	12	12	12
Cutting speed *v_c_*, m/min	1000	1000	200	1000	1200	1200	1200	200
Feed per tooth *f_z_*, mm/tooth	0.1	0.1	0.02	0.1	0.02	0.02	0.02	0.02
Rotational speed *n*, rpm	12,732	12,732	3979	12,732	23,873	23,873	23,873	3979
Number of passes *i*	2	2	1	2	1	9; 1 *	9	1

* Final pass.
